# The effect of hyperarticulation on speech comprehension under adverse listening conditions

**DOI:** 10.1007/s00426-021-01595-2

**Published:** 2021-09-26

**Authors:** Jayanthiny Kangatharan, Maria Uther, Fernand Gobet

**Affiliations:** 1 Brunel University London, Kingston Lane, London, Uxbridge, UB8 3PN UK; 2 University of Wolverhampton, Wulfruna Street, Wolverhampton, WV1 1 LY UK; 3 London School of Economics and Political Sciences, London, WC2A 2AE UK

## Abstract

Comprehension assesses a listener’s ability to construe the meaning of an acoustic signal in order to be able to answer questions about its contents, while intelligibility indicates the extent to which a listener can precisely retrieve the acoustic signal. Previous comprehension studies asking listeners for sentence-level information or narrative-level information used native listeners as participants. This is the first study to look at whether clear speech properties (e.g. expanded vowel space) produce a clear speech benefit at the word level for L2 learners for speech produced in naturalistic settings. This study explored whether hyperarticulated speech was more comprehensible than non-hyperarticulated speech for both L1 British English speakers and early and late L2 British English learners in quiet and in noise. Sixteen British English listeners, 16 native Mandarin Chinese listeners as early learners of L2 and 16 native Mandarin Chinese listeners as late learners of L2 rated hyperarticulated samples versus non-hyperarticulated samples in form of words for comprehension under four listening conditions of varying white noise level (quiet or SNR levels of + 16 dB, + 12 dB or + 8 dB) (3 × 2× 4 mixed design). Mean ratings showed all three groups found hyperarticulated speech samples easier to understand than non-hyperarticulated speech at all listening conditions. Results are discussed in terms of other findings (Uther et al., 2012) that suggest that hyperarticulation may generally improve speech processing for all language groups.

## Introduction

In noiseless environments, speakers of a second language (L2) perform like native speakers in speech perception tasks (e.g. Nábělek & Donahue, 1984). However, when background noise is present, their speech perception in L2 is more affected than in their first language (L1) (Florentine, 1985a, 1985b; Garcia Lecumberri & Cooke, 2006; Mayo et al., 1997; Takata & Nábělek, 1990). This effect has been suggested to be associated with listeners’ age of L2 acquisition (Scott, 1994), the time period of L2 study (Florentine, 1985a, 1985b) and the environmental situation under which listening occurs (Takata & Nábělek, 1990).

In the presence of noise, non-native speakers’ performance on L2 speech perception tasks, such as when listening to sentences in babble noise (Florentine, 1985a, 1985b, 1985c), has been shown to depend on the age at which they acquire L2 (Florentine, 1985b; Mayo et al., 1997). For example, research by Florentine (1985b) revealed that exposure to L2 from infancy onwards, rather than only after puberty, helped L2 listeners to perform as well as L1 speakers on speech perception tasks in the presence of increasing noise. These data are interpreted as indicating a sensitive period after which learning a second language negatively affects L2 listeners’ perception of L2 in noise (Florentine, 1985b). It was shown that in speech perception tasks with noise, early learners of L2 performed better and benefitted more from sentence-level contextual information compared to late but very proficient L2 learners, indicating that late L2 listeners will have difficulty perceiving L2 in noise even with extensive exposure.

However, early L2 learners’ ability to perceive L2 in noise has been suggested to be inferior to and qualitatively different from that of native listeners’ due to L1 experience (Mayo et al., 1997). Because L1 English listeners had higher noise-tolerance levels than early L2 English learners (Mayo et al., 1997), L1 listeners have been claimed to be able to recover quickly from noise-induced disturbance because of their linguistic knowledge of established L1 categories (Bradlow & Alexander, 2007).

As a result of late L2 listeners’ limited exposure to L2, it has been argued that late L2 listeners do not respond to clear speech as well as early L2 learners or L1 listeners (Bradlow & Bent, 2002; Smiljanić & Bradlow, 2011). Specifically, clear speech is considered to have signal enhancements such as slow speech rate and broad pitch range that all listeners are regarded to be able to access (Hazan & Simpson, 1998). However, clear speech also includes subtle enhancements that are specific to the target language and that are considered to improve the acoustic distance among phonologically different contrasts in the target language (Bradlow & Bent, 2002; Smiljanić & Bradlow, 2011). It is therefore considered that only L1 listeners and early L2 learners, who are familiar with the difference in duration between short and long vowels in English, will be able to show sensitivity to and thus benefit from an exaggerated dissimilarity between these vowels in clear speech (Bradlow & Bent, 2002).

Evidence for late L2 listeners’ limited benefit from clear speech, as compared to conversational speech under degraded situations comes from Bradlow and Bent (2002) who aimed to find out if L2 listeners with low proficiency can benefit from clear speech produced by L1 English speakers under different noise conditions. In that study, in which slow speech rate, broad pitch range and larger sound pressure levels were considered aspects that lead to the improved signal of clear speech, late L2 listeners showed a smaller benefit from clear speech compared to L1 listeners. This outcome has been suggested to be caused by late L2 listeners’ limited experience with the L2 sound structure. The authors, therefore, argued that the nature of clear speech is not oriented towards L2 listeners but towards L1 listeners (Bradlow & Bent, 2002). However, one has to note that clear speech in their study was produced by instructing L1 speakers to read sentences as if talking to hearing-impaired listeners. Clear speech in that study was therefore not elicited in natural interaction with a real interlocutor, and it was not specifically aimed at L2 speakers.

There is an abundance of literature on the effects of clear speech and intelligibility (for detailed information, see Bradlow & Bent, 2002; Bradlow et al., 2003; Kangatharan, 2015). It has been suggested that the cognitive resources that we assign to our semantic processing of speech can help us to predict upcoming words to support our lexical understanding (Schiller et al., 2008**)**. These predictive mechanisms that our brain uses to process speech can enhance our ability to better understand speech. Because speech with foreign accents is more difficult to process than speech with no accents, it could be argued that providing semantic context could help the perception of speech that was produced by foreign-accented L2 speakers. This was found by an eye-tracking study, in which listeners did not depend on the acoustic input that the speakers provided to help them select a picture because they relied on semantic information (Lev-Ari, 2015). They particularly made use of interpretations from the context when the listeners received the information from foreign-accented L2 speakers. This finding raises the question as to whether, in the absence of semantic context, hyperarticulated speech provides sufficient acoustic information to aid in the processing of speech for both L1 and L2 listeners.

Previous research has also suggested a more shallow semantic activation in listeners when they heard speech in a foreign accent compared to speech with no accent (Romero-Rivas et al., 2016), indicating that foreign-accented speech and native-accented speech are processed differently. However, more recent research revealed that foreign-accented speech affected understanding only during the early stage of speech processing while there was no difference in listeners’ processing of native and foreign-accented speech at the later stage (Schiller et al., 2020). This implies that listeners’ overall understanding was not influenced by the presence or absence of an accent. Based on these findings, it could be speculated that hyperarticulated speech would help remove any differences in early-stage speech processing for both L1 and L2 listeners.

There is little research indicating which clear speech properties are beneficial for L1 and L2 listeners’ speech comprehension under noisy conditions. Previous studies mainly looked at the relationship between intelligibility and several clear speech properties at vowel level, and highlighted the role of expanded vowel space in enhancing vowel intelligibility (Ferguson & Kewley-Port, 2002, 2007). Intelligibility of speech does not equal speech comprehension (Hustad & Beukelman, 2002): comprehension assesses a listener’s ability to construe the meaning of an acoustic signal to be able to answer questions about its contents, while intelligibility indicates the extent to which a listener can precisely retrieve the acoustic signal (Hustad, 2008). It is notable that previous comprehensibility studies that asked listeners for sentence-level information (Hustad & Beukelman, 2002) or narrative-level information (Hustad, 2008) were presented to native listeners. There was one study examining potential differences in processing hyperarticulated clear phonemes in native and non-native (Greek) speakers of English (Uther et al., 2012). In that study, there was an enhancement of the response to the phonetic change in both language groups at a pre-attentive level, suggesting the automatic processing of hyperarticulated phonemes was equivalent in both groups, whereas there was evidence of brain indices of attentional switch in Greek speakers that was not there for native English speakers. However, there has been no research on whether clear speech properties (e.g. expanded vowel space) produce a clear speech benefit at the word level for L2 learners.

Thus, the goal of the current study was to determine whether expanded vowel space improves clarity for listeners in quiet and in noise conditions at the word level. This would help evaluate whether hyperarticulated speech is beneficial to both native and non-native listeners and therefore contributes to an enhanced understanding of speech in the English language. Based on previous research (Bradlow & Bent, 2002; Ferguson & Kewley-Port, 2007, Smiljanić & Bradlow, 2011) it was hypothesised that expanded vowel space leads to speech that is more comprehensible than normal speech for both L1 British English speakers’ and early and late L2 British English learners under quiet and adverse listening conditions. This would be in line with the Hyper-and Hypoarticulation (H&H) theory according to which adults modify their speech to maximise discriminability to provide the listener with sufficient information to make speech comprehension possible (Lindblom, 1992).

## Methods

This study had two parts: a speech production experiment to elicit spontaneous speech produced when doing a ‘Spot the Difference’ task with different types of interlocutors, and a listening experiment using target words extracted from the recordings of the first experiment.

## Speech production experiment

### Design

This experiment used a 2 (interlocutor’s accent: native, foreign) × 2 (interlocutor’s physical appearance: native, foreign) × 3 (three target vowels: /a:/, /uː/ and /iː/) mixed design. Therefore there were four different types of interlocutors: NLNS (native looking and native sounding), NLFS (native looking and foreign sounding), FLNS (foreign looking and native sounding), and FLFS (foreign looking and foreign sounding). The interlocutor’s accent and physical appearance were between-subject variables, and the target vowels were a within-subject variable. The dependent variable was the extent of hyperarticulation in the target words in which one of the three target vowels was present.

### Materials and apparatus

For the purpose of eliciting the tense target vowels /a:/, /uː/ and /iː/, the words ‘car’, ‘blue’ and ‘beach’ were chosen as specific target words to get the vowel area data. To facilitate the elicitation of these target vowels from the native speakers, three “Spot-the-difference” (Diapix) tasks were used. These tasks were modified versions of the tasks developed by Baker and Hazan (2011). The first picture depicted a beach scene, the second a farm scene and the third a street scene (see Appendix). A digital voice recorder Edirol R-09HR by Roland (sampling rate: 44.1 kHz) was used to record all verbal interactions. Each interaction was recorded as a mono 16-bit file in wav format.

Initially, 150 target words belonging to one of three target vowels were recorded from native speakers during the completion of the Diapix task. The vowels /a:/, /i:/, /u:/, /i/, /e/ and /ɒ/ were chosen from the target words “car”, “beach”, “blue”, “pink”, “red” and “shop” as they contained a minimum of one sample. Five instances from each of the three vowels /a:/, /i:/ and /u:/ were taken randomly from each of the four experimental conditions as expressed by different speakers. In addition, instances from the three vowels /i/, /e/ and /ɒ/ were taken randomly from each of the four experimental conditions as distractors.

### Participants

The participants who produced the speech samples were fifty-two female White British speakers aged between 18 and 35 years. They were asked to communicate with one individual from four different speaker groups to complete a Diapix task. The four groups were: (a) White British speakers, (b) speakers of White European ethnicity with native White British appearance and foreign accent, (c) speakers of Asian (Indian/Pakistani or Bengali) ethnicity with foreign appearance and native accent, and (d) speakers of Asian ethnicity with foreign appearance and foreign accent. Participants were recruited from the student population of Brunel University. This study was approved by the Ethics committee of the Psychology Department at Brunel University.

### Procedure

#### Recording procedure with native speakers during the Diapix task

In each 30-min audio-recorded interaction, a White British English speaker and an interlocutor were seated opposite each other. Each participant received a folder with three pictures, each illustrating a different scene. For each scene, there were 13 differences between the picture that one participant received and the picture of their partner interlocutor. The differences included an absent object or an alteration to one of the objects on the picture. Participants were instructed to work together to verbally find out the differences between their pictures. The task lasted about ten minutes for each of the three pictures. Participants filled in consent form prior to participating and were debriefed following participation.

## Listening experiment

### Design

The design of the listening experiment was a 3 × 2 × 4 × 3 mixed design, with the vowels (/a:/, /i:/, /u:/), recipient condition (native sounding and foreign sounding), and noise levels (quiet vs. + 8 dB SNR vs. + 12 dB SNR vs. + 16 dB SNR) representing the within-subjects variables and the three listening groups (native listeners, early non-native listeners, and late non-native listeners) representing the between-subjects variable.

### Materials and apparatus

After the recordings were generated, target words were extracted from the sound files. Word-length target files were equated for root-mean-square amplitude before being mixed with white noise as background noise generated in MATLAB (similar to Billings et al., 2009) at + 16 dB, + 12 dB and + 8 dB SNRs. The noise created for each target word had the same total duration as the speech signal. White noise was employed because this type of energetic masking was found to influence native and non-native listeners to the same degree for everyday words and syntactically and semantically simple speech material (Cutler et al., 2004; Garcia Lecumberri et al., 2010). This type of noise is not specific to speech and thus represents environmental degradation of speech. Based on previous research, the SNR at + 8 dB SNR was chosen as medium noise, and the SNR at 12 dB SNR was selected as low noise, with + 16 dB SNR chosen as a very low noise level (Bradlow et al., 2003; Cutler et al., 2008). This means that at 16 dB SNR more signal is presented than noise, and a person will hear more speech than noise at 16 dB SNR than one will at 12 dB SNR or 8 dB SNR. Similarly, one will have more signal than noise at 12 dB SNR than at 8 dB SNR.

Stimuli were presented on a computer in an experimental cubicle using e-prime software (Schneider et al., 2002a, 2002b) via headphones (Sennheiser HD429) at a comfortable listening volume. Participants responded by using the computer keyboard. Responses were automatically recorded for each participant.

### Participants

The listeners consisted of three groups: (a) 16 monolingual speakers of British English (aged 18–45 years) from the Southeast London area; (b) 16 native speakers of Mandarin Chinese (aged 18–45 years) who learned English before the age of twelve years; and (c) 16 native speakers of Mandarin Chinese (aged 18–45 years) who learned English after the age of twelve years. The average age of twelve was chosen based on prior research (Flege, 1995; Flege & MacKay, 2004). Non-native listeners were recruited from Brunel University’s Language Centre. All listeners were enrolled at Brunel University and had no speech or hearing impairments at the time of testing. Participants were paid £10 for participating. The study was approved by the Ethics committee of the Psychology Department at Brunel University.

### Procedure

#### Speech comprehension task: rating procedure with native and non-native listeners

In the speech comprehension task participants listened to 480 audio stimuli via headphones set at a comfortable listening level prior to the task starting. To minimize learning effects over the time-length of the study, the order of presentation of the word stimuli was randomised. The order of the stimuli was also randomized across SNR levels. This randomization varied from listener to listener. The session lasted approximately 40 min. In this task, participants were asked to listen to each word stimulus with care and then to indicate on a scale from 1 to 6 to what extent the stimulus was easy to understand (1 = not easy to understand at all; 6 = very easy to understand). There was a 500 ms delay in presenting subsequent stimuli after the participant indicated their response. The presentation of the next word was signaled by an arrow that was displayed for 200 ms.

Before the experimental session, a practice session with 16 trials was implemented in which four non-experimental practice words were presented at one of the four SNR levels so that listeners became accustomed to the nature of the task and the stimuli with noise. None of the experimental target words were used for this practice session. During the experimental session, each word stimulus was presented three times for each noise level and listeners could take as long as necessary to give a response.

## Results

### Speech production study

A mixed ANOVA (2 × 2 × 3 mixed design) was used to analyze the effects of appearance and accent on vowel triangle area and it showed that accent significantly differed across conditions (*F* (2, 40) = 61.698; *p* < 0.05; *η*^*2*^*p* = 0.755). There was no main effect of appearance. There was no significant accent by appearance interaction.

**Fig. 1:**
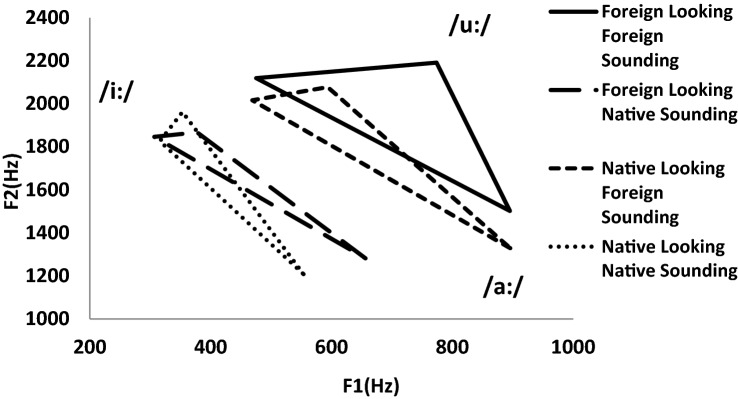
Areas of target vowels in foreign-looking foreign-sounding condition, native-looking foreign-sounding condition, native-looking native-sounding condition and foreign-looking native-sounding condition

**Fig. 2:**
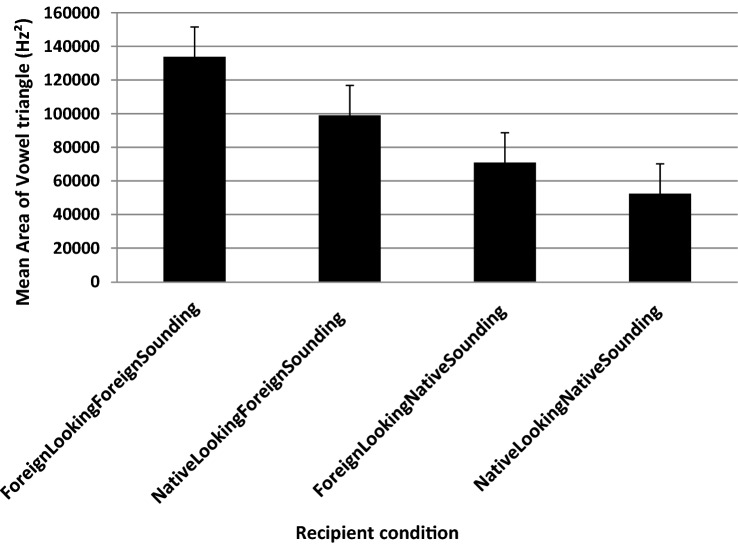
Mean area calculated from the vowel triangle in F2/F1 space in the foreign-looking foreign-sounding condition, foreign-looking native-sounding condition, native-looking foreign-sounding condition and native-looking native-sounding condition

The vowel triangles with the formant frequencies from the vowels of the target words ‘car’, ‘blue’ and ‘beach’ are shown in Fig. 1. The mean areas from the vowel triangles are shown in Fig. 2.

A comparison between the foreign-sounding conditions and the native-sounding conditions revealed that the vowel space was significantly larger for the foreign-sounding conditions than the native-sounding conditions. This indicates an acoustic exaggeration of vowels in a speech to foreign-accented L2 speakers irrespective of whether their appearance is native or foreign. This finding indicates that native speakers hyperarticulate vowels in speech to interlocutors who require linguistic clarifications, such as foreign-sounding interlocutors compared to native-sounding interlocutors irrespective of their appearance.

## Listening experiment

**Fig. 3:**
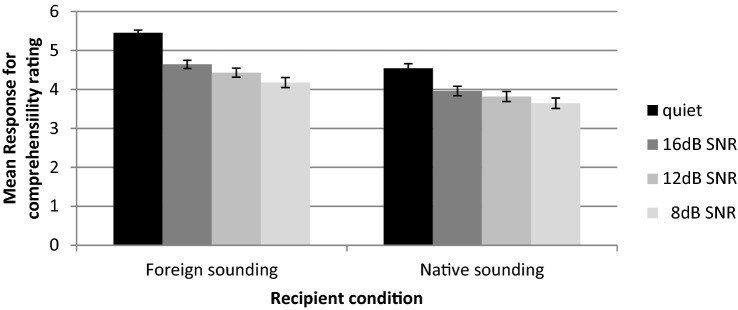
Mean rating for comprehensibility ratings of speech to different recipient conditions (foreign-sounding; native-sounding) at different SNRs (quiet, + 16 dB, + 12 dB and the + 8 dB). Error bars show ± 1 standard errors from the mean

A mixed ANOVA across all three listening groups showed that speech to foreign-sounding interlocutors was easier to understand than speech to native sounding interlocutors (*F* (1, 45) = 205.002; *p* < 0.05, *η*^*2*^_*p*_ = 0.820) (Fig. 3). Glass et al. (1972) showed that the F-test is very robust to violations of the interval data assumption; therefore, it is appropriate to use ANOVA with responses in a Likert format. This supports the hypothesis that hyperarticulated speech will improve listeners’ comprehension.

The assumption of sphericity was violated for noise with a Mauchly’s W of 0.041 (*p* < 0.001). According to the Greenhouse–Geisser correction, the effect for noise is significant (*F* (1.2, 53.99) = 118.76; *p* < 0.001, *η*^*2*^_*p*_ = 0.725). This is supported by Multivariate Tests, which do not follow such an assumption showing a significant main effect across groups (*F* (3, 43) = 43.325; *p* < 0.05, *η*^*2*^_*p*_ = 0.751) (Fig. 3). Pairwise comparisons showed that while speech in silence was easier to understand than at + 16 dB SNR (*t* (47) = 10.545, *p* < 0.0083,* r* = 0.797), at + 12 dB SNR (*t* (47) = 10.677, *p* < 0.0083,* r* = 0.734), and at + 8 dB SNR (*t* (47) = 11.427, *p* < 0.0083,* r* = 0.678), speech at + 16 dB SNR was easier to understand than at + 12 dB SNR (*t* (47) = 7.019, *p* < 0.0083,* r* = 0.981) and + 8 dB SNR (*t* (47) = 9.886, *p* < 0.0083,* r* = 0.958), while speech at + 12 dB SNR was easier to understand than at + 8 dB SNR (*t* (47) = 8.287, *p* < 0.0083,* r* = 0.981). These results show that speech under quiet conditions or in low noise levels is easier to understand than speech in higher noise levels across groups. There were no other significant main effects, except for vowels, *F* (2, 45) = 16.280; *p* < 0.05, *η*^*2*^_*p*_ = 0.268. Accordingly, ‘car’ was easier to understand than’beach’ and ‘blue’, and ‘beach’ was easier to understand than ‘blue’. This observed vowel effect could be due to the vowel /a/ being articulated with more pronounced lip opening and emission of sound compared to the vowels /i/ and /u/. Accordingly, the resulting naturally more intense sound of /a/ could have contributed to this observed vowel effect for /a/ in this study although it has to be acknowledged that it could also have got to do with vowels differing in sound pressure level and ‘intrinsic pitch’ (IF0) (Whalen & Levitt, 1994).

There was no significant effect on the listener group. There were no significant interactions, except for a significant interaction between recipient condition and noise: listeners’ rating evaluated speech to foreign-sounding interlocutors as more comprehensible than speech to native sounding interlocutors, with this effect being stronger in the quiet condition than the other conditions (*F* (3, 43) = 8.693; *p* < 0.05, *η*^*2*^_*p*_ = 0.378) (Fig. 3). Thus, this result shows that not only at quiet but also in the presence of noise, stimuli with expanded vowel space were rated more comprehensible across listener groups than stimuli without expanded vowel space. This indicates a role of hyperarticulation in improving comprehension of speech that is presented in background noise.

## Discussion

This study aimed to answer the research question as to what effect vowel space expansion has on L1 and L2 listeners’ comprehensibility of speech. First, the investigation of mean rating revealed across listener groups that speech directed at foreign-sounding interlocutors was easier to understand than speech directed at native-sounding interlocutors. It was also observed that speech at quiet and low noise levels was easier to understand than speech at high noise levels. These findings are consistent with the hypothesis that hyperarticulated speech with expanded vowel space can improve listeners’ comprehension.

It is interesting to note that the comprehension scores in the quiet condition were not closer or even identical to 6 as one could have expected a ‘ceiling’ effect in that condition. One could speculate that despite the very small vocabulary size and good listening conditions, the comprehension scores in the quiet condition did not reach a perfect 6 because speech in interaction to native sounding interlocutors can be considered to be casual speech in which coarticulation and vowel and consonant reduction could be seen as having led to reduced comprehension in contrast to speech produced in interaction to foreign-sounding interlocutors.

Similarly, comprehension scores in speech produced in interaction to foreign-sounding interlocutors can be speculated to not be closer to 6 because it was generated in a dynamic, spontaneous interaction with the aim of jointly solving a task, which means that speech will be not as comprehensible as if it had been produced in dialogue with foreign-sounding interlocutors that is expressed in a deliberate and direct way, or if it has been produced in form of speech that is read out loudly.

There were no differences in performance at quiet or at the different noise levels across listener groups. These observations do not support previous research that proposed that at quiet early L2 learners would show a speech comprehension benefit from expanded vowel space that is comparable to that of L1 English listeners and that is larger than that of late L2 English learners (Bradlow & Bent, 2002; Smiljanić & Bradlow, 2011). Similarly, these observations do not support suggestions by previous research that in noise early L2 English learners will, in comparison to late L2 English learners, find stimuli with expanded vowel space more comprehensible but less than L1 English speakers (Florentine, 1985a, 1985b; Garcia Lecumberri & Cooke, 2006; Mayo et al, 1997; Takata & Nábělek, 1990). Thus, it seems that despite their varying proficiency levels in L2, both early and late L2 listeners appear to have equally benefitted from the stretched vowel space that was embedded in natural-speech to foreign-sounding interlocutors. These observations seem to suggest that at quiet and in noise, vowel hyperarticulation can assist with listening comprehensibility for both L1 listeners, and early and late L2 learners of English.

This finding, therefore, appears to suggest that L2 listeners’ recognition of words in English can be supported through vowel hyperarticulation. This result supports previous findings according to which vowel hyperarticulation was proposed to likely lead to increased comprehensibility of speech (Ferguson & Kewley-Port, 2007). The result also seems to confirm that vowel hyperarticulation, if elicited in a communicative setting, can lead to improved speech comprehensibility of words (Ferguson & Kewley-Port, 2007). The present experiment can therefore be seen as extending studies that showed that clear vowel hyperarticulated speech can lead to higher speech intelligibility (e.g. Bond, Moore & Gable, 1996; Hazan & Markham, 2004; Johnson et al, 1993). The observation that speech to foreign-sounding interlocutors was easier to understand than speech to native sounding interlocutors at quiet and at different noise levels supports the H&H theory according to which adults modify their speech to maximize discriminability to provide the listener with sufficient information to make speech comprehension possible (Lindblom, 1992).

As noted in the introduction, previous research found that providing semantic context assists the processing of speech that was produced by L2 speakers with foreign accents because listeners were observed to depend on semantic information instead of the acoustic input that the speakers provided to help them choose a picture (Lev-Ari, 2015). This reliance on top-down processing to semantically process speech was particularly observed when the listeners received the information from foreign-accented L2 speakers. The current study appears to indicate that, in the absence of a social context, hyperarticulated speech provides sufficient acoustic information from the bottom up to assist both L1 and L2 listeners with different levels of proficiency in their processing of speech at the word level.

An event-related potential (ERP) study on word integration revealed semantic N400 effects in Spanish listeners for sentences that were generated by both native and non-native speakers of Spanish (Romero-Rivas et al., 2016). This semantic integration of words into context, with listeners being able to predict upcoming words and their semantic characteristics, can be considered to have led to listeners’ enhanced perception of speech that was produced by native speakers and by non-native speakers with accents (Romero-Rivas et al., 2016). Nonetheless, it was noted that the level of semantic activation appeared more superficial when listeners heard the sentences in a foreign accent, implying a differential processing of native-accented and foreign-accented speech. To address this point and investigate the aspect of anticipation directly, a recent ERP study was conducted that aimed to provide more insight into whether foreigner-accented speech reduces or increases anticipation (Schiller et al., 2020). It used a within-participants design and monitored brain-activity before the presentation of the critical word.

The study reported an early ERP difference in the processing of native and foreign-accented speech, probably because listening to sentences with a very predictable lexical item produced by a foreign-accented speaker decreased the brain’s anticipatory processes. This lack of early brain activity, suggesting that there was no word anticipation, was indicated by the absence of phonological mismatch negativity (PMN) in foreign-accented speech. By contrast, later ERP components did not reveal any significant difference between native and foreign-accented speech processing, implying that listeners’ overall performance was not affected depending on whether they listened to sentences in native or non-native accents (Schiller et al., 2020). This shows that foreign-accented speech only affected the early stages of speech processing and there was no difference in the understanding of native and foreign-accented speech at a later stage. In relation to the results of the current study, it appears that in comparison to non-accented speech, foreign-accented speech is more difficult to process, whereas both L1 and L2 speakers do not show any differences in processing when speech is hyperarticulated.

The present study does not uphold prior research that reported early L2 listeners have higher noise-tolerance levels than late L2 listeners (Mayo et al., 1997). This finding can therefore be considered to disagree with the previous finding that when their speech perception in L2 was interrupted by noise, late L2 listeners’ perception of speech in L2 was more affected than their speech perception in L1 (Florentine, 1985a, 1985b; Garcia Lecumberri & Cooke, 2006; Mayo et al., 1997; Takata & Nábělek, 1990). This lack of a higher speech comprehension benefit for early L2 learners as compared to late L2 listeners might have been due to the limited nature of the task in the present experiment as it employed a listening comprehension task (Munro & Derwing, 1999). The absent finding of higher noise-tolerance levels for early L2 listeners might, therefore, be accounted for by the limited speech material available and the simplicity of target words used. The limited statistical power of the experiment can also be seen as a weakness of this study. Psychology studies being underpowered is an issue in the field of psychology (Maxwell, 2004). Accordingly, future research could recruit more volunteers for each participant condition and use a different speech comprehensibility task with speech material that are not restricted to simple target words.

Another reason for the absent finding of a higher speech comprehension benefit for early L2 learners than late L2 listeners might be due to the confound of the length of experience using the language between the early and late L2 learner groups in the L2 country. Consequently, even if early and late L2 learners might have started L2 acquisition at a different age, the difference in the length of their exposure to L2 might have contributed to this result. However, it can be argued that this confound is inevitable because even if early and late L2 learners are matched for the length of experience using L2 and differ in age of L2 acquisition, early L2 learners might have been exposed to more L2 when watching news or television programs in L2 in their native country compared to late L2 learners, and vice versa. Nonetheless, this result can be used by future experiments investigating the effect of age of L2 acquisition on L2 learners’ performance on L2 comprehension tasks to look at additional factors that might lead to a difference in their performance between early and late L2 learners such as L2 learners’ reported percentage use of L1 and the number of speakers they interact with in L1 on a regular basis (Flege & MacKay, 2004).

Similarly, the data did not show that early L2 learners’ comprehensibility was lower than that of L1 English speakers. This is inconsistent with previous research in which L1 listeners were reported to recover more quickly than early non-native L2 listeners from adverse listening conditions due to their established L1 categories (Mayo et al., 1997). In addition, the data do not support previous research, which showed that L1 listeners experienced a perceptually higher benefit than late L2 learners because of late L2 learners’ insufficient experience in the L2 sound structure (Bergman, 1980). Thus, it cannot be argued that late L2 learners’ L1 might have affected their performance in the listening comprehensibility rating as they may have perceptually assimilated incoming L2 phonemes to L1 categories (Best & Tyler, 2007). Nonetheless, it has been suggested that late L2 learning does not prevent the perception of L2 vowels that functionally is similar to native-like perception of L2 vowels (Flege & MacKay, 2004).

The findings of this study appear to confirm the role of speech to foreign-sounding interlocutors to be of didactic benefit. The findings of this study, therefore, suggest that vowel space expansion as it is used together with other acoustic–phonetic features in speech to foreign-sounding listeners might be useful in linguistic training programs to facilitate foreign-sounding listeners’ comprehension of the target language.

Overall, the findings also emphasise the importance of knowing the target language, which can lead to the dissolution of any differences in speech intelligibility between different groups of listeners. For example, in the area of forensic speech science, it has been shown that knowing the language used by an incriminated voice sample presents an advantage when it comes to recognizing voices. For example, previous research has shown that both German listeners and English listeners who were knowledgeable about German as the target language performed better at voice identification in German than English listeners who were not knowledgeable about German (Köster et al., 1995). Moreover, it was shown that compared to Spanish and Chinese listeners without any knowledge of German as the target language, Spanish and Chinese L2 speakers of German performed better in identifying a German voice. However, Spanish and Chinese L2 speakers of German performed worse compared to German native speakers and English L2 speakers (Köster & Schiller, 1997). While speech in Köster and Schiller’s (1997) study was not hyperarticulated, the results of the present study appear to indicate that the differences in speech intelligibility between different groups of listeners could be removed when speech is hyperarticulated. The results of the current study, therefore, show they can be applied to forensic speech science research.

Future research could address the aforementioned weaknesses of the current study by exploring the effect of hyperarticulation on native and non-native listeners’ speech comprehension by using different vowel samples that were elicited in a natural and spontaneous speech setting. Further research could also make use of noise levels other than used in the current study, and use different types of noise to address the questions how hyperarticulation might support speech perception and comprehension when speech is degraded at word and sentence level. In light of recent studies (e.g. Redford, 2014) that highlighted the connection between clarity and speech rate, it would also be relevant to understand how presenting hyperarticulated speech stimuli at different speech rates influences speech perception and whether it would help with speech comprehension.

It also has to be noted that in the current study, the vowel samples that were presented in the speech comprehension task included repetitions of word stimuli and were not all first-mentioned forms of the word stimuli or were not all new referents. However, it has been shown that speech stimuli that are produced repeatedly in a natural conversation are generated with less acoustic importance than novel referents (Prince, 1981; Watson et al., 2010). It can, therefore, be considered that because the speech stimuli used in the speech comprehension task included second-mentioned forms of the speech stimuli, this could have likely led to the production of words with less extreme vowels (Pettinato et al., 2016). This in turn could have impaired the perceptual effects of hyperarticulated stimuli and could have been reflected by the lack of differential effects on the perception by native and non-native listeners of English.

In conclusion, this study addressed the research question of the effect vowel space expansion has on L1 and L2 listeners’ comprehensibility of speech. Across all listener groups (early L2 learners of English, late L2 learners of English and L1 English speakers), speech at the word level to foreign-sounding interlocutors was easier to understand than to native sounding interlocutors at both quiet and all noise levels. It therefore seems that vowel hyperarticulation used together with other acoustic–phonetic features in speech to foreign-sounding listeners has an enhancing effect on the comprehensibility in foreigner-directed speech (FDS). Although this study appears to indicate that vowel hyperarticulation could be used as a linguistic instrument for didactic purposes, there are some limitations to consider such as the simplicity of the speech material used and the limited statistical power of the experiment, which would need to be addressed by future research.

## Appendix

‘Beach’ picture (A).

‘Beach’ picture (B).

‘Farm’ picture (A).

‘Farm’ picture (B).

‘Street’ picture (A).

‘Street’ picture (B).
